# Understanding health disparities affecting people of West Central African descent in the United States: An evolutionary perspective

**DOI:** 10.1111/eva.13549

**Published:** 2023-05-03

**Authors:** Anthony R. Mawson

**Affiliations:** ^1^ Department of Epidemiology and Biostatistics, School of Public Health, College of Health Sciences Jackson State University Jackson Mississippi USA

**Keywords:** adaptation, African Americans, chronic disease, evolution, genetics, health disparities, health profile, liver, parasitemia, structural racism, tropical diseases, vitamin A

## Abstract

Human populations adapting to diverse aspects of their environment such as climate and pathogens leave signatures of genetic variation. This principle may apply to people of West Central African descent in the United States, who are at increased risk of certain chronic conditions and diseases compared to their European counterparts. Less well known is that they are also at reduced risk of other diseases. While discriminatory practices in the United States continue to affect access to and the quality of healthcare, the health disparities affecting African Americans may also be due in part to evolutionary adaptations to the original environment of sub‐Saharan Africa, which involved continuous exposure to the vectors of potentially lethal endemic tropical diseases. Evidence is presented that these organisms selectively absorb vitamin A from the host, and its use in parasite reproduction contributes to the signs and symptoms of the respective diseases. These evolutionary adaptations included (1) sequestering vitamin A away from the liver to other organs, to reduce accessibility to the invaders; and (2) reducing the metabolism and catabolism of vitamin A (vA), causing it to accumulate to subtoxic concentrations and weaken the organisms, thereby reducing the risk of severe disease. However, in the environment of North America, lacking vA‐absorbing parasites and with a mainly dairy‐based diet that is high in vA, this combination of factors is hypothesized to lead to the accumulation of vA and to increased sensitivity to vA as a toxin, which contribute to the health disparities affecting African Americans. vA toxicity is linked to numerous acute and chronic conditions via mitochondrial dysfunction and apoptosis. Subject to testing, the hypothesis suggests that the adoption of traditional or modified West Central African‐style diets that are low in vA and high in vA‐absorbing fiber hold promise for disease prevention and treatment, and as a population‐based strategy for health maintenance and longevity.

## 1 INTRODUCTION

Human populations adapting to diverse aspects of their environment such as climate and pathogens leave signatures of genetic variation. This principle may apply to people of West Central African descent in the United States, who are at increased risk of certain vascular, metabolic, malignant, and allergic diseases compared to their European counterparts. However, they are also at reduced risk of death from other diseases, notably malaria, dengue hemorrhagic fever, yellow fever, and nonalcoholic fatty liver disease. While discriminatory practices and poorer access to healthcare and other resources continue to affect the health of African Americans, this paper presents the hypothesis that disparities in health outcomes affecting African Americans are also due in part to evolutionary physiological adaptations to the ancestral environment. This environment involved continuous exposure to the vectors of endemic tropical diseases. The most lethal of these, the malaria parasite *Plasmodium falciparum*, along with other parasitic and helminthic organisms, selectively absorb vitamin A (vA), a potentially toxic fat‐soluble molecule, from the human host. The use of vA in parasite reproduction also contributes to the signs and symptoms of the respective parasitic diseases. The proposed evolutionary adaptations included (1) sequestering vA away from the liver, where it is mainly stored, to other organs, to reduce its accessibility to *Plasmodia*, and (2) slowing the metabolism and catabolism of vA, causing it to accumulate to subtoxic concentrations, thereby weakening the parasite and reducing the risk of severe disease. These adaptations were apparently successful in enhancing survival in the original environment. However, in the North American environment, characterized by the absence of vA‐absorbing parasites and by a dairy‐based diet high in vA, it is hypothesized that these adaptations lead to its overall accumulation, rendering people of West Central African descent particularly sensitive to vA as a cellular toxin. Exposure to and the accumulation of vA are proposed to explain the increased susceptibility to organ‐specific diseases that were rare in the original environment and are increasingly linked to vA toxicity via mitochondrial dysfunction and apoptosis. Subject to testing, the vA toxicity hypothesis suggests that the adoption of traditional or modified West Central African‐style diets, which are low in vA and high in vA‐absorbing fiber, could help to prevent and treat these diseases. They could also serve as a population‐based strategy for maintaining overall health and increasing longevity.

## 2 HEALTH DISPARITIES AFFECTING AFRICAN AMERICANS

**FIGURE 1:**
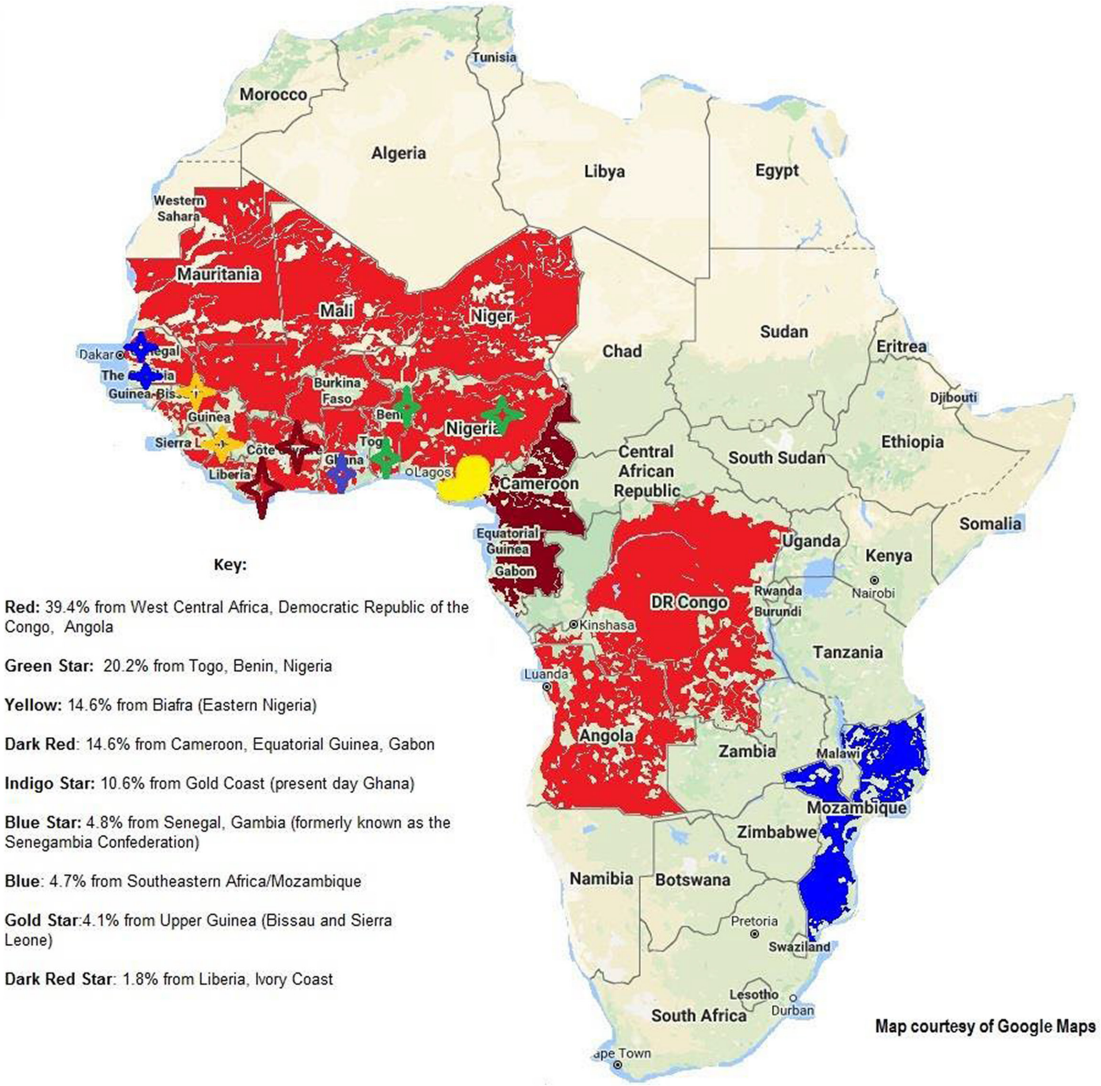
Ancestral origins of African Americans.

The approximately 39 million Americans of African ancestry now living in the United States, mostly from forced relocation over preceding centuries, originated from countries in West and Central Africa; these included the Congo and Angola (39%); Togo, Benin, and Western Nigeria (20%); East Nigeria (15%); and the Cameroons, Equatorial Guinea, and Gabon (15%) (Fortes‐Lima, 2015). (Figure 1). For reasons that are not fully understood, African Americans (AA) are at greater risk of morbidity and mortality than Americans of European descent from cancers of the blood and bone marrow, breast, lung, ovary, prostate, colon, and rectum (Long et al., 2013; Smedley et al., 2008); metabolic and cardiovascular diseases including hypertension, type 2 diabetes, kidney disease, obesity, gout, congestive heart failure, and stroke (American Heart Association, 2015; Kumar & Lenert, 2016); eczema (American College of Allergy, 2015; Fischer et al., 2017; Silverberg, 2015); asthma (United States Environmental Protection Agency, 2015); and allergies to eggs and milk (Cuatrescases et al., 1965; Wegienka et al., 2012). These conditions were once rare in African countries themselves (Maiyaki & Garbati, 2014). Yet today, the prevalence of hypertension in AA men and women is over 40%, among the highest in the world, occurring earlier in life and of greater severity than among other groups (American Heart Association, n.d.). African Americans are also at risk of certain genetic diseases and conditions, including sickle cell anemia—the most common blood disorder in the United States—and glucose‐6‐phosphate dehydrogenase deficiency (G6PDd), which is found almost exclusively among males in malaria‐endemic regions (Beutler & Duparc, 2007).

Life expectancy is shorter for AAs compared to European Americans (EAs). Although life expectancy at birth increased for both males and females from 1980–2014, it was 6.9 years longer for EA males than for AA males and 5.6 years longer for EA females than for AA females (Centers for Disease Control and Prevention, n.d.‐a). The infant mortality rate for AA infants (deaths in infants up to 12 months of age per 1000 live births) is more than double that of EA infants, due to higher rates of stillbirth, birth defects, low birth weight, premature birth and Sudden Infant Death Syndrome (Centers for Disease Control and Prevention, n.d.‐b; Bryant et al., 2010; March of Dimes, 2015). Rates of maternal mortality among AAs are also over twice those of women of European descent (CDC, 2018a).

In sub‐Saharan Africa, death rates from cancer, diabetes and heart disease are expected to exceed those from infectious diseases by 2030; 46% of Africans suffer from high blood pressure, the highest rate worldwide, and a growing percentage of people are overweight (World Health Organization, 2016). Even in the mid‐1990s the prevalence of diabetes exceeded that of the white population in Durban, South Africa (Omar et al., 1993). Yet less than a century earlier, coronary heart disease was rare in many parts of Africa (Walker & Sarell, 1997), as it was in western populations before World War I (McCrae, 1912). Cancer rates in Africa were also low (Walker, 1995).

## 3 STRUCTURAL RACISM AND HEALTH DISPARITIES

Health disparities are differences in disease incidence, prevalence, mortality, morbidity (complications), survivorship, including quality of life after treatment, burden of disease, and stage at diagnosis (National Cancer Institute, n.d.). Disparities in health care affecting AAs are generally attributed to structural racism, which has created limited access to healthcare, poorer quality of healthcare, education, and housing, as well as stressful experiences related to racial discrimination, and lower overall incomes. The continuing existence of discrimination, stress, and poorer healthcare is well‐documented (Assari et al., 2017; Institute of Medicine, 2002; LaVeist & Isaac, 2013). Williams et al. (Williams et al., 2019) have proposed that socioeconomic factors alone do not account for racial and ethnic inequities in health and that racism is a fundamental cause of adverse health outcomes for racial and ethnic minorities. For each of the primary domains of racism affecting mental and physical health outcomes—structural racism, cultural racism and individual‐level discrimination—Williams et al. present data and suggest priorities for future research to advance knowledge in the area.

The legacy of 400 years of slavery, violent oppression, discrimination, and emotional stress suffered by African Americans, and the struggle for access both to receive and provide health care, continue to have pervasive effects (Braveman & Gottlieb, 2014; Colen et al., 2017; deShazo, 2018). The COVID‐19 pandemic in 2020–2022 also disproportionately affects African Americans; across the United States, COVID‐19 mortality rates in predominantly black counties have been sixfold higher than in predominantly white counties (Yancy, 2020).

African Americans are also at increased risk of diabetes and its complications after controlling statistically for socioeconomic status (Williams, Priest, et al., 2016). Non‐Hispanic black women have rates of preterm birth that are 40% higher than those of Hispanic and non‐Hispanic white women after adjustment for maternal socioeconomic status and education (Behrman & Butler, 2006). Chronic worry about experiencing racial discrimination is reported to affect risks of premature birth among AA women, possibly through neuroendocrine, vascular or immune mechanisms involved in responses both to stress and the initiation of labor (Dominguez, 2011). Such worry has been proposed to explain the puzzling observation of greater disparities in preterm birth among more socioeconomically advantaged AA women (Braveman et al., 2017).

While disparities in access to and the quality of health care affecting AAs remain rooted in structural racism and discrimination, the overall health profile of people of West Central African descent suggests a role for additional factors. For instance, in a 25‐year follow‐up study on the health of medical graduates, black compared to white physicians had a higher risk of cardiovascular disease, with an earlier onset, twofold higher rates of diabetes and hypertension, a 40% higher rate of coronary artery disease, and a much higher case‐fatality rate of 52% versus 9% (Thomas et al., 1997). Similarly, in a diverse cohort of 927 youth with type 1 diabetes, AAs compared to other groups had a higher glycated hemoglobin (HbA1c) level after adjustment for many factors, higher rates of severe hypoglycemia and ketoacidosis, higher required doses of insulin per body weight, and a higher rate of hypertension. AA participants also had less residual β‐cell function, which likely contributed to a worsened prognosis for type 1 diabetes (Redondo et al., 2018). Higher overall HbA1c levels are reported in AA children with type 1 diabetes compared to white children, after adjustment for socioeconomic status, diabetes duration, and insulin dose (Bell et al., 2009; Delamater et al., 1991).

After being diagnosed with type 1 diabetes, AA youth have unexplained adverse outcomes of earlier onset and prognosis for long‐term complications.

A study of hypertension among West Africans in Africa and their descendants reported a stepwise increase in hypertension as groups moved from rural to urban Africa to the Caribbean, and from the Caribbean to the US. People of African descent in the US had hypertension levels that were twice as high as those in Africa, and higher than among mainly white populations in Europe (Cooper et al., 1997; Cooper & Rotimi, 1997). Another study comparing hypertension rates in West Africa, the Caribbean and the US, based on 10,014 individuals in seven locations and using standardized data collection methods, reported a consistent gradient of hypertension prevalence, rising from 16% in West Africa to 26% in the Caribbean and to 33% in the United States. Mean blood pressures were similar among those aged 25 to 34, but the increase in rates of hypertension with age was twice as steep in the US as in Africa. Age‐specific mortality rates from chronic diseases were often several‐fold higher in younger black adult age‐groups overall than in most high‐income countries. Even more surprising, age‐specific mortality rates from chronic diseases are higher in sub‐Saharan Africa than in virtually all other regions of the world, both in men and women (de‐Graft Aikins et al., 2010).

These findings pose the following questions: How does the North American environment contribute to increased rates of chronic disease in all population groups that have been studied? Why are African Americans more susceptible to these diseases than EAs? And, why are chronic disease rates rising rapidly in Africa when they have historically been low? In short, why were Africans healthy in their original environment but unhealthier than Europeans in North America, and increasingly in Africa itself? In what way were Africans well‐adapted health‐wise in the West Central African environment, but fare less well in the United States? This paper suggests answers to these questions.

## 4 HEALTH DISPARITIES IN THE REVERSE DIRECTION

A major clue to understanding health disparities affecting AAs is that such disparities also exist in the reverse direction; that is, people of West Central African descent are protected from severe outcomes and are less likely than EAs to die from other diseases, notably malaria, yellow fever, and dengue hemorrhagic fever (Goucher et al., 1998; Halstead et al., 2001; Kiple & King, 1981; McGuire & Coelho, 2011; Sierra et al., 2007). They are also at lower risk of nonalcoholic fatty liver disease (NAFLD) (Guerrero et al., 2009; Pan & Fallon, 2014), defined as hepatic fat accumulation (steatosis) >5% of total weight of the liver. NAFLD affects over 30% of older EAs (Dongiovanni et al., 2013). AAs also have less hepatic lipid content, lower plasma lipoprotein lipase, and less overall liver disease (Sumner et al., 2010). NAFLD is a growing global epidemic, affecting millions of people worldwide. In the US, Hispanics are the most disproportionately affected ethnic group and AAs the least affected by hepatic steatosis and elevated aminotransferase enzyme levels. The burden of NAFLD is widely underestimated (Sherif et al., 2016). Considering the vital role of the liver in metabolism and in overall health, the anomaly of lower rates of NAFLD in AAs suggests an underlying biological process or mechanism related to the liver which potentially explains the pattern of health disparities affecting AAs, as outlined below. African Americans also have the lowest risk of osteoporosis of any racial group at younger ages, as well as higher bone densities and lower bone turnover rates (Overfield, 1995, p. 71). AA males have half the nonvertebral fracture rate of EAs (Griffin et al., 1992; Weinstein & Bell, 1988). But when hip fracture does occur, AA women are more likely to die than EA women; they also have longer hospital stays and are less likely to be ambulatory at discharge (Cauley, 2011).

The arguments that follow propose that differences in health outcomes affecting AAs are due in part to evolutionary physiological adaptations to the West Central African environment. This environment is broadly characterized by exposure to potentially lethal or disabling diseases resulting from parasitic organisms, notably malaria, schistosomiasis, and onchocerciasis (Cummings & Turco, 2009). These adaptations were successful in enhancing survival in the original environment but are potentially disadvantageous in the environment of North America, as explained below.

About 3.3 billion people are at risk of malaria due to *Plasmodium falciparum*, the most dangerous malaria parasite, making it one of the world's most important health problems (Crawley et al., 2010). There were 214 million new cases of malaria worldwide in 2015, with Africa accounting for most (88%), and an estimated 438,000 deaths from malaria worldwide, mostly in Africa (90%) and in children under age 6, that is, about 0.2% of the 214 million cases, indicating the comparative rarity of lethal outcomes (WHO, 2018).

*Schistosoma mansoni*, a waterborne flatworm trematode parasite, is the leading cause of schistosomiasis and the second most common human parasitic disease after malaria. It is found in areas with water contaminated by freshwater snails, the intermediate host for the parasites. The disease causes liver and intestinal damage. In 2016, 206.5 million people in 75 countries had the disease and about 200,000 people, mostly in Africa, died from it (Global Burden of Disease, 2016).

The helminth nematode worm *Onchocerca volvulus* causes onchocerciasis (“River blindness”), a disease transmitted by repeated bites from blackflies. Infection results in severe itching and skin atrophy and in visual impairment that can progress to blindness. Until recently about 18 million people were affected by the disease, but it has been greatly controlled in affected communities by long‐term application of the drug ivermectin (Otabil et al., 2019).

## 5 ENVIRONMENTAL EXPOSURE TO VITAMIN A‐INGESTING PATHOGENS

A second major clue to understanding the health profile of people of West Central Africa is that both *P. falciparum* (Mizuno et al., 2003) and *Onchocerciasis volvulus* (Stürchler et al., 1981) selectively absorb vitamin A from the human host. Vitamin A (vA) is a major potential toxin. The uptake of vA by the malaria parasite also correlates with the number and concentration of parasites in the blood (Mizuno et al., 2003). In the case of trematode parasites, including *Schistosoma japonicum*, the major active vA metabolite retinoic acid (RA) is actively taken‐up by the parasitic nematode *Brugia Malaya* (Wolff & Scott, 1995), and vA signaling pathways are involved (Qiu et al., 2013). It is not known if Schistosoma selectively absorb vA. However, significant reductions in serum vA, zinc, and carotenoids were observed in patients with active *Schistosoma mansoni* infection and complications of the disease, including anemia (Mikhail & Mansour, 1982), suggesting that the parasite selectively absorbs vA from the host.

Use of the ingested vA by the parasite in reproduction is hypothesized to contribute to the signs and symptoms of the respective diseases (malaria, schistosomiasis, and onchocerciasis), representing endogenous forms of hypervitaminosis A (vA poisoning; Mawson, 2013; Mawson et al., 2017; Mawson & WaKabongo, 2002). Over the course of millennia, the people of West Central Africa adapted physiologically to the toxic effects of vA, resulting from its uptake and use by the parasites. The proposed pathogenesis of malaria is first outlined, followed by a summary of the biochemistry of vA.

## 6 THE PATHOGENESIS OF MALARIA

Malaria parasites spend more than a week in the host liver before invading the red blood cells (RBCs; Milner, 2018). My hypothesis is that the merozoite‐stage parasites, emerging from the liver packed with vA, use it as a cell membrane destabilizer to invade and reproduce in the RBCs (Mawson, 2013). As the damaged RBCs begin to die, their contents of vA are released into the circulation, causing signs and symptoms of the disease as an endogenous form of hypervitaminosis A. The features of malaria include fever, flu‐like illness, fatigue, gastrointestinal symptoms, respiratory distress, anemia, and jaundice. These features closely resemble those of vA toxicity. Obesity is a major risk factor for malaria (Wyss et al., 2017) and may increase susceptibility to the disease due to the accumulation of vA in the liver. This hypothesis of the pathogenesis of malaria has been confirmed by the results of a study comparing vA profiles in patients infected with *Plasmodium vivax* (PV, *n* = 74) with those of nonmalarial patients with fever (*n* = 25). The PV patients had significantly lower serum levels of retinol but higher levels of retinoic acid, as predicted (Na et al., 2020).

## 7 RETINOIDS

**FIGURE 2:**
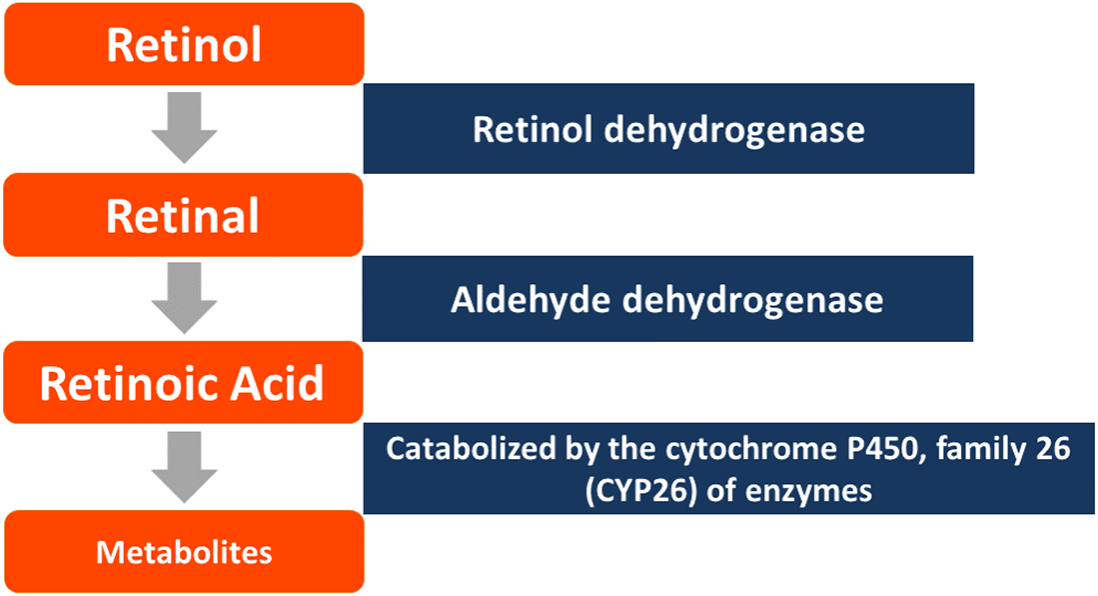
Metabolism of vitamin A.

Retinoids are natural and synthetic forms of vA and are considered essential in very low concentrations for numerous biological functions. In higher concentration, they are toxic to cells as well as mutagenic and teratogenic (D'Ambrosio et al., 2011; Li et al., 2014). Serum retinol concentrations are homeostatically controlled by the liver but are not closely correlated with clinical signs. They are therefore not useful for determining the vA status of individuals or the impact of interventions (WHO, 2011). Retinoic acid (RA), the main active metabolite, is produced from retinol via hydrolysis of retinyl esters (REs) in the liver (see Figure 2). Retinol is delivered to the target tissues bound to protein, as retinol‐binding protein4 (RBP4), and oxidized to retinal (retinaldehyde) via alcohol dehydrogenase. RA is synthesized from retinaldehyde via an aldehyde dehydrogenase reaction and exerts its numerous effects by controlling the expression of over 500 genes through binding to and activating the nuclear protein receptors: retinoic acid receptors (RARs) and retinoid X receptors (RXRs; Theodosiou et al., 2010).

About 80% of vA is stored in the liver—in quantities sufficient to last the average adult about 2 years without the need for additional intake (De Luca & Creek, 1986)—as well as in the lung, adipose tissue, and intestine. It can be toxic if released into the circulation unbound to protein, as retinyl esters or retinoic acid. Toxicity can result from excessive dietary intake, dietary supplements, food supplemented with vA, and vA medications, and also when the liver becomes saturated with vA and stored retinyl esters (REs) spill into the circulation. REs destroy cell membranes and are a major source of vA toxicity.

RA is up to 100 times more biologically active and hence more toxic than retinol. Samples obtained from cadavers have suggested that 7.5% of total serum vA as REs is diagnostic for toxicity at the individual level in adults (Olsen et al., 2018). Growing evidence suggests that the molecular basis of retinoid toxicity involves mitochondrial dysfunction and cell death via apoptosis (de Oliveira, 2015; Melnik, 2017).

Acute vA toxicity is characterized by fever, nausea and vomiting, headache, fatigue, blurred vision, lack of muscular coordination, skin peeling, and mitochondrial dysfunction. Early symptoms of chronic vA toxicity are alopecia, dry eyes, severe headache, and generalized weakness. Later changes include cortical hyperostosis of bone and arthralgia, fractures, pruritus, failure to thrive, hepatomegaly and splenomegaly (de Oliveira et al., 2012; Olson et al., 2022; Penniston & Tanumihardjo, 2006). Intakes of vA only marginally above the recommended amount in early pregnancy (7800 μg/day) are associated with congenital malformations (Allen & Haskell, 2002). Zambian children with a high prevalence of hypervitaminosis A, recruited into an intervention study that included vA at the recommended level as retinyl palmitate or a placebo for 90 days, showed that bone formation was strongly improved in the placebo group consuming low preformed vA: this suggested that chronic consumption may cause hypervitaminosis A and impair bone formation in children (Tanumihardjo et al., 2019).

A variety of infections are associated with transient declines in plasma retinol concentrations, giving the appearance of a deficiency state. However, experimentally induced *Escherichia coli* mastitis in heifers was associated with increased fever and decreased serum retinol, but also by a significant increase in all‐trans‐retinoic acid. These changes in vA concentration profiles during infection argue against a deficiency state and instead indicate a profound shift in retinoid metabolism towards the active metabolite (Merris et al., 2004). As an earlier commentator noted: “… people seem reluctant to recognize just how toxic is vitamin A. Considered as a chronic poison, vitamin A is probably more harmful than cyanide … Vitamin A is an essential substance … But it is a very dangerous substance and it needs to be handled with care.” (Pitt, 1979).

## 8 ADAPTATIONS TO VITAMIN A‐INGESTING ENDEMIC PARASITES

It is hypothesized that over the course of millennia the people of West Central Africa adapted physiologically to the threat of lethal parasitic infection in at least two ways: (1) sequestering vA away from the liver to other tissues, to reduce its accessibility to the parasite and (2) reducing the enzymatic metabolism and catabolism of vA, causing it to accumulate to sub‐toxic levels and weaken the parasite, thereby reducing the potential lethality of the disease. Evidence in support of the hypothesis is summarized below.

1. AAs and EAs differ in fat distribution in abdominal adipose tissue, which contains 10–20% of total retinoids in the body as retinyl esters (Frey & Vogel, 2011; Wolf, 1998). EAs have an increased visceral abdominal adipose tissue (VAT) mass compared to AAs, whereas AAs have a greater subcutaneous abdominal adipose tissue (SAT) mass compared to EAs (Hoffman et al., 2013). Aldehyde dehydrogenase and retinaldehyde dehydrogenase catalyze the first and second steps in the oxidation of retinol to retinal and retinoic acid, and both have been identified in adipose tissue (Li et al., 2014). Stellate (vA‐containing) storage cells are also present in the kidneys and lung (Blomhoff et al., 1990). Differences between African Americans and European Americans in the distribution and concentration of vA in other tissues await investigation.
2. AAs have a reduced rate of fat and fatty acid metabolism than EAs (Sharp et al., 2002). The capacity of skeletal muscle to oxidize fatty acids in obese AAs and EA women was 25% lower in AA women compared to EA women (*p* < 0.05); mitochondrial and microsomal acyl‐CoA synthetase (ACS) were also significantly lower in AA women (*p* < 0.005), and it was suggested that decreased fatty acid oxidation by skeletal muscle could contribute to the maintenance of obesity (Privette et al., 2003). African Americans also have lower rates of CYP2D6‐ and CYP2C19‐dependent drug metabolism compared to EAs (Aklillu et al., 2002). The cytochrome P450 (CYP) 26 enzymes are specifically involved in the catabolism of RA to oxidized metabolites and are highly expressed in the liver, small intestine, and mitochondrial membranes (Napoli, 2000). The availability of RA is regulated by a balance between the synthesizing enzymes (retinaldehyde dehydrogenases‐RALDHs) and the catabolizing CYP26 enzymes. Much is known about the function of RALDHs during development, but little is yet known about the role of the CYP26 enzymes and the hypothesized differences between AAs and EAs.

The observation that serum vA concentrations are lower in AAs than in EAs, specifically in AA women (Ballew et al., 2001; Hanson et al., 2016), is usually interpreted as indicating low vA nutritional status or deficiency in AA women (plasma retinol concentration < 0.70 μmoL/L or <20 μg/dL) (WHO, 2009). However, it may instead reflect lower liver stores of vA and reduced mobilization and secretion of vA as a characteristic feature of people of West Central African descent. The finding could also mask important but unknown differences between AAs and EAs in retinyl esters and retinoic acid.

The mainly plant‐based diets of West Central Africa are low in retinyl esters (Haskell, 2012) and high in vitamin A‐absorbing fiber and could contribute to lowering the potential toxicity of vA from its use by the malaria parasite. In the United States, children and teenagers derive >80% of their vA from animal sources and the average adult derives >65% (US Department of Agriculture, 2014), whereas in Uganda the comparable figures are 5%–25% for preschool children and 5%–20% for adults (Hotz et al., 2012).

In areas where malaria is infrequent, other diets evolved that depended on prevailing conditions and resources. The Masai people of Kenya and northern Tanzania are nomadic herdsman living at a high altitude where mosquitos are typically absent. Their diet consists mostly of milk, meat, and blood, and little carbohydrate. Despite their high fat and cholesterol diet associated with a high intake of vA, the Masai traditionally had low blood cholesterol levels, low rates of cholesterol gallstones, low blood pressure and low rates of atherosclerotic coronary artery disease (Biss et al., 1971; Ho et al., 1971; Mann et al., 1964). This suggests a genetic basis (Wagh et al., 2012), with the Masai able to tolerate a high intake of vA with less toxicity.

## 9 UNDERSTANDING HEALTH DISPARITIES AFFECTING PEOPLE OF WEST CENTRAL AFRICAN DESCENT IN THE UNITED STATES

**FIGURE 3:**
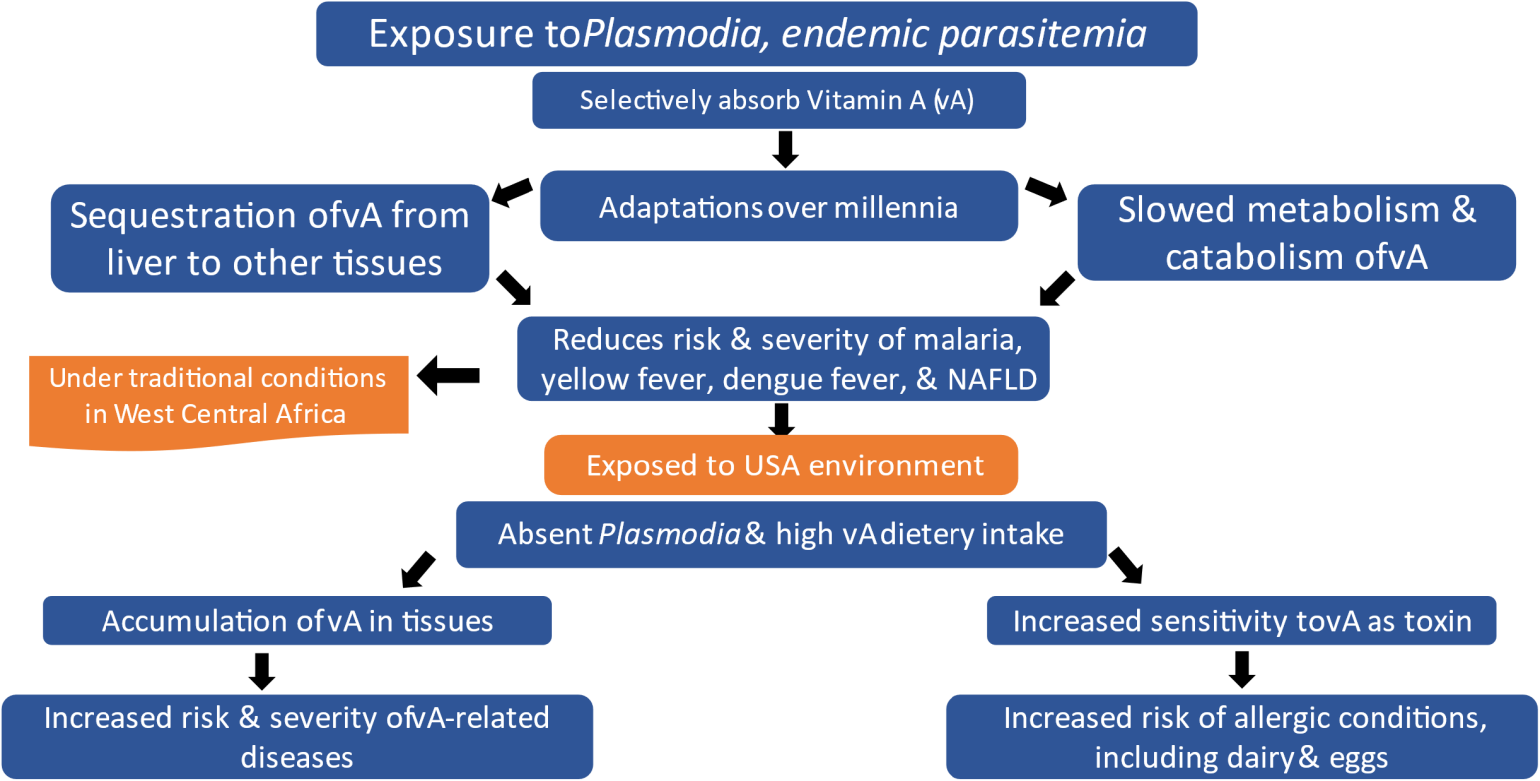
Theory of the health profile of people of West Central African descent. Malaria parasites selectively absorb vitamin A and spend over a week in the liver, the main storage site of vA. Endemic exposure to Plasmodia and other common parasites in the original environment is hypothesized to have led to evolutionary adaptations involving (1) the sequestration of vA from the liver to subcutaneous adipose and other tissues, to reduce its accessibility to the parasite; and (2) reduced vA enzymatic metabolism and catabolism, causing it to accumulate to sub‐toxic concentrations and weaken the parasite, thereby reducing the risk of severe disease. These adaptations may have reduced the severity of malaria, yellow fever, dengue, and the risk of NAFLD in people of West Central African descent. However, in North America, where major parasites are absent and high vA‐containing diets are the norm, these adaptations may cause vA to accumulate in the tissues, thereby increasing the risk and severity of many chronic diseases. They may also have led to an enhanced sensitivity to vA as a cellular toxin, and increased risks of allergy. These adaptations may be contributing to the emergence of a similar health profile in West Central Africa.

The evolutionary adaptations described above were apparently successful in the environment of sub‐Saharan Africa, serving to reduce the severity and lethality of endemic parasitic infections. However, in the environment of North America they are potentially disadvantageous, for two reasons:

1. vA‐absorbing parasites are lacking, and malaria is no longer endemic in the United States. Before the 1950s, malaria was common throughout the southeastern US, but screening of housing, improved socioeconomic conditions, environmental management, and vector‐control efforts during the late 1940s, especially use of the insecticide DDT, succeeded in interrupting malaria transmission (Andrews et al., 1950). Malaria parasitemia and the absorption of vA by the merozoites could have a role in regulating vA nutritional status (Mizuno et al., 2003; SanJoaquin & Molyneux, 2009; Stoltzfus et al., 1989), but this conjecture awaits further investigation.
2. In recent decades, AAs have adopted the same dairy‐based diet as that of EAs (Tibbs et al., 2001). Compared to Africans, AAs consume significantly more protein, meat, saturated fat, cholesterol and vA (O'Keefe et al., 2007). My hypothesis is that the absence of vA‐absorbing parasites and adoption of the dairy‐based diet of the US, coupled with the proposed physiological adaptations, leads to an overall accumulation of vA, rendering people of West Central African descent particularly sensitive to vA as a cellular toxin and mutagen. This increased exposure to and accumulation of vA could explain the increased rates of diseases that were once rare in the original environment. The vA‐toxicity hypothesis of the health profile of people of West Central African descent is depicted in Figure 3, below.

## 10 DISPARITIES INVOLVING ORGAN‐SPECIFIC DISEASES

Rates of colorectal cancer in AAs are dramatically higher than in Africans (60/100,000 vs. <1/100,000), and slightly higher than in EAs (Williams, White, et al., 2016). AAs also have higher death rates from cancer of the breast, cervix, ovary and lung, and a twofold higher rate of triple‐negative breast cancer, a more aggressive type (Pickle et al., 1996). AA males in 2014 also had the highest incidence rate of prostate cancer (Shenoy et al., 2016) and were more likely to die of the disease than any other group (CDC, 2018b). Consistent with the overall model, the risk factors for prostate cancer include high dietary intakes of vitamin A (Hanks et al., 1993). Higher dairy milk consumption is also associated with a 50 percent increased risk of breast cancer, contrary of current guidelines for dairy milk consumption (Fraser et al., 2020). The death rate for all cancers combined is 25 percent higher for AAs than for EAs (American Cancer Society, 2018). AAs also have the highest death rates from stroke (Shenoy et al., 2016) and are twice as likely to have Alzheimer's disease and other dementias as EAs (Barnes & Bennett, 2014), possibly related to increased concentrations of vA in the brain.

African Americans have a higher percentage of abdominal subcutaneous adipose tissue and a lower percentage of abdominal visceral fat (Hoffman et al., 2013; Rønn et al., 2020). They also have lower rates of NAFLD than those of other ethnic groups (Schneider et al., 2013), and significantly lower triglyceride levels than Hispanics and EAs (Foster et al., 2013). These differences in disease rates may be related to increased concentrations of vA in the organs in question in AAs compared to EAs, and increased accumulation due to reduced metabolism and catabolism. Yet AAs who do develop NAFLD are overrepresented in terms of disease precursors to CVD, including obesity, hypertension, and diabetes, and are more prone than EAs to develop advanced liver disease. In the Jackson Heart Study, a cohort of over 5000 older AAs, participants with high levels of abdominal visceral and liver fat had high rates of hypertension, diabetes, and dyslipidemia (Liu et al., 2011). Fatty liver was also associated with coronary atherosclerotic calcification independent of abdominal visceral fat or Body Mass Index (Liu et al., 2012). Other studies indicate a strong association between NAFLD and atherosclerotic calcification (Lee et al., 2018).

Growing evidence implicates retinoids in the fundamental processes relevant to atherosclerosis, including vascular calcification and endothelial function. Mice fed excess vitamin A for 12 months developed heart valve stenosis and leaflet calcification, suggesting that hypervitaminosis A is a risk factor for calcific aortic valve disease (Huk et al., 2013). The effects of retinoids on lipid metabolism and adipogenesis may indirectly cause inflammation and atherosclerosis (Rhee et al., 2012). However, AAs are typically less likely to develop coronary atherosclerotic calcification and have markedly lower levels than EAs when they do develop it (Divers et al., 2017), possibly related to their low risk of osteoporosis, higher bone densities, and lower bone turnover rates (Overfield, 1995).

What could explain the paradoxical observations that AAs are at increased risk of metabolic syndrome despite lower levels of liver fat, and prone to advanced liver disease? The postulated tendency toward reduced vA metabolism and hence increased vA accumulation could account for these facts. Even with lower levels of liver fat, AAs may be more sensitive to vA as a toxin and more likely to experience inflammatory changes, for example, in the liver, indicated by increased liver enzymes, as well as more prone to obesity, hypertension, and diabetes, and to severe manifestations and secondary consequences of these conditions. In partial support of this hypothesis, a 12‐year prospective study of liver enzymes and diabetes risk in the Atherosclerosis Risk in Communities (ARIC) study, involving 7495 EAs and 1842 AAs without diabetes found that high liver enzyme levels were independently associated with diabetes risk. The enzyme gamma‐glutamyl transferase (GGT) was most strongly related to diabetes, even at levels considered within the normal range (≤60 U/L). Adjusted incidence rates of diabetes by quartiles of liver enzymes were similar in terms of gender but higher in AAs. These findings have suggested that abnormal liver enzymes precede the diagnosis of diabetes by many years, and that individuals with elevated liver enzymes even within supposedly normal limits are at high risk for diabetes, especially AAs (Schneider et al., 2013).

**FIGURE 4:**
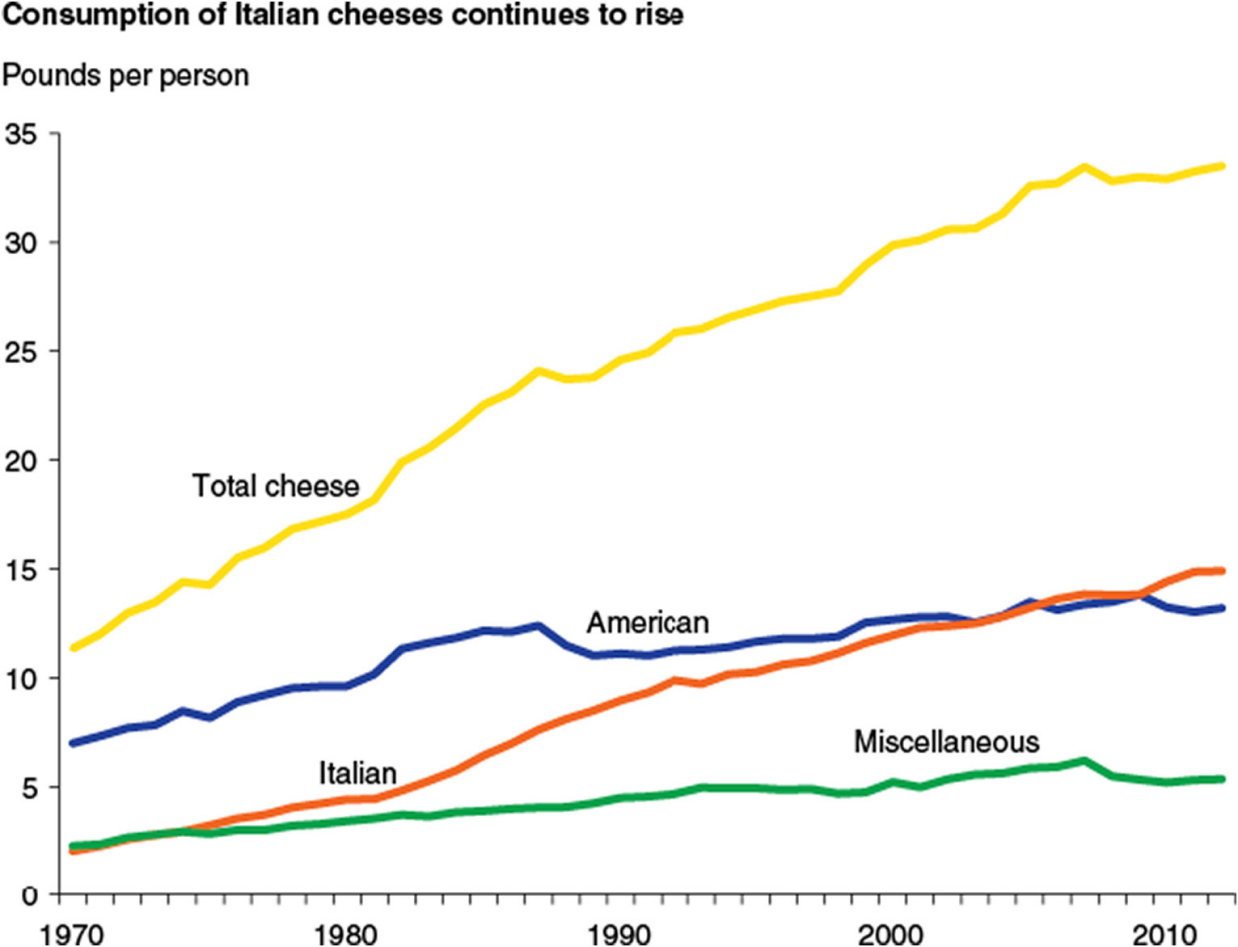
Consumption of cheese in the United States, 1970–2010.

In summary, the increased susceptibility of people of West Central African descent to certain chronic diseases, including diabetes, from which they were originally protected, may be due to the adoption of vA‐rich North American diets and to an increased sensitivity to vA as a toxin, resulting from its slower metabolism and catabolism and greater accumulation. Of special interest is the observation that widening disparities in heart disease mortality rates have occurred only since the 1970s. In Mississippi before that time, available records suggest that differences in CVD death rates between AAs and EAs were less marked or even nonexistent. AAs had similar rates to those of EA Mississippians and Americans in general, and AA males had lower CVD death rates than either white Mississippi males or white US males (Cosby et al., 2006; Jones et al., 2000). In the 1980s the magnitude of the disparity between AAs and EAs in CVD mortality rates increased dramatically. Yet during that time, race‐linked factors such as discrimination and limited access to health care were either static or improving. Other factors to consider include changes in occupational opportunities and to dietary influences favoring processed and fast‐food consumption (Cosby et al., 2006), including foods fortified by or supplemented with vA. Cheese consumption has increased in recent decades, as has vA‐fortified low‐fat milk. Cheese availability was 11.4 lbs in 1970 and 33.5 lbs/person in 2012, nearly three times higher. Consumption of frozen pizza, macaroni and cheese, and prepackaged cheese has also increased (USDA [US Department of Agriculture], 2014; see Figure 4).

In 1908, it was observed that rabbits fed meat, milk and eggs developed arterial lesions like those of atherosclerosis in humans (Ignatovski, 1908). However, the association between dairy consumption and heart disease‐related clinical outcomes remains controversial (Drouin‐Chartier et al., 2016), partly because the traditional focus on fat, cholesterol, and calcium as risk factors may have obscured the role of vA itself. Research is needed to assess vA intake (including food and vitamin supplements) directly and to determine its association with various health outcomes in AAs, EAs and other groups.

## 11 SENSITIVITY TO VITAMIN A AND RISK OF ALLERGIES

The vA toxicity theory suggests that higher rates of eczema and asthma among AAs compared to EAs (Gupta et al., 2016) may be due to increased concentrations of vA in the skin and lung and reduced rates of vA metabolism, and hence greater sensitivity to vA as a toxin. Use of topical retinoids for skin of color is associated with irritant contact dermatitis, leading to increased risks of postinflammatory pigmentation and keloid or hypertrophic scarring, and recommendations to start at a lower concentration and titrate upwards (Davis & Callender, 2010). The use of isotretinoin, a synthetic retinoid, for AA patients with recalcitrant nodulocystic acne, was associated with an early onset flare in areas of the face that were devoid of lesions prior to treatment (Kelly & Sampson, 1987).

Retinoid toxicity is also linked to bronchial asthma (Mawson, 2001; Na et al., 2017; Solt et al., 2010). Intakes of vA (≥2.5 times the recommended daily amount) during pregnancy were associated with increased risks of asthma in school‐age children in Norway. Most mothers used cod liver oil, in which the median intake of supplemented vA was ≥300 μg/d compared to the standard daily dose of 250 μg (Parr et al., 2018). Therapeutic retinoids are frequently linked to irritant contact dermatitis (Veraldi et al., 2015). The proposed hypothesis is that AAs are more sensitive to vA as a toxin than EAs and have a higher concentration of vA in affected tissues. Higher rates of allergic reactions to milk (lactose intolerance) (Bailey et al., 2013) and eggs are reported in Africans and AAs compared to EAs (Gray et al., 2016; Wegienka et al., 2012), and 70%–80% of AAs are lactose intolerant (National Institutes of Health, 2009). The high content of vA in eggs and in dairy products such as milk and cheese (Glasper et al., 2011) could explain the disparities in question. Lower rates of alcohol use among AAs compared to EAs (Thomas & Price, 2016) may reflect the fact that alcohol consumption increases the secretion of vA from the liver and enhances its toxicity (Leo & Lieber, 1999).

## 12 REDUCED VITAMIN A METABOLISM

Observing that red blood cell disorders such as sickle cell anemia and the thalassemias were highly prevalent in tropical areas where malaria was endemic, the geneticist J.B.S. Haldane hypothesized in the 1940s that natural selection had created biological traits that protect individuals from severe malaria (Haldane, 1949). Haldane's hypothesis was confirmed by A. C. Allison, who showed that the geographical distribution of the sickle‐cell mutation in the beta hemoglobin gene (HBB) was limited to Africa and correlated with malaria endemicity, and individuals who carried the sickle‐cell trait were resistant to malaria (Allison, 1954). A striking worldwide geographical difference also exists for a mutation in the Duffy antigen gene (FY), which encodes a membrane protein used by the *Plasmodium vivax* malaria parasite to enter red blood cells. This mutation disrupts the protein, conferring protection against *P. vivax* malaria, and is pervasive throughout sub‐Saharan Africa but is rare outside of Africa (Sabeti, 2008).

The origin of these traits and understanding the manifestations of the disorders have remained uncertain, but they can be tentatively explained by the second hypothesized evolutionary adaptation to endemic parasitism proposed here: that the reduced metabolism and catabolism of vA cause it to accumulate to toxic concentrations and weaken the parasites, thereby reducing the risk of severe disease. Over an evolutionary timescale, these suggested mutations in the metabolism of vA could also account for the unique health profile of people of West Central African descent. This profile includes sickle cell anemia (SCA) and glucose‐6‐phosphate dehydrogenase deficiency (G6PDd). Both sickle cell trait and G6PDd are protective against death from cerebral malaria.

SCA occurs in about 1 in 500 live births and affects 90,000 to 100,000 people in the US, mainly African Americans. The alteration of a single nucleotide in the gene for the beta chain of the hemoglobin protein, hemoglobin S (HbS), results in rigid, sickle‐shaped red blood cells that typically appear around 6 months of age. SCA is associated with attacks of severe pain, swelling of the extremities, bacterial infections, and increased risks of kidney disease, retinopathy and stroke (NHLBI, n.d.). Sickle cell trait affects 1–3 million Americans, 8–10% of African Americans, and > 100 million people worldwide. Unlike sickle cell disease, where two genes, one from each parent, cause the production of abnormal hemoglobin, individuals with sickle cell trait carry only one defective gene and typically have few or no medical problems (American Society of Hematology, 2018).

G6PDd is an X‐linked recessive inborn error of metabolism that predisposes to hemolysis in response to stress and consumption of certain foods, notably fava beans. Symptoms such as jaundice, dark urine, shortness of breath, and fatigue may develop, but most people with the condition are asymptomatic and many never have symptoms. G6PDd is the most common human congenital defect of metabolism, defined by decreased NADPH+H(+) and a reduced form of glutathione. It is associated with susceptibility to Alzheimer's disease (Evlice & Ulusu, 2017) and contributes to the pathophysiology of cerebral vasculopathy in children with SCA (Joly et al., 2016).

Recalling that the retinoid‐converting enzymes include retinol dehydrogenase, alcohol dehydrogenase, aldo‐keto reductase, and aldehyde dehydrogenase (Hong et al., 2015), the mechanism underlying the health profile of people of West Central African descent may involve an overall reduction of dehydrogenase enzyme activity such that, in the dietary environment of North America, it could lead to an accumulation of vA and its noted adverse health outcomes. The incidence of osteoporosis and related fractures in African American women is half that of Caucasian women, but an exponential increase in hip fracture rates occurs in African American women over age 70, with those sustaining osteoporosis‐related fractures experiencing increased disability and decreased survival.

Consistent with the hypothesis of lower dehydrogenase enzyme activity, mice deficient in retinaldehyde dehydrogenase (which converts retinaldehyde to retinoic acid) have a higher trabecular and cortical bone mass compared to age‐ and sex‐matched control mice; they also have increased cortical bone thickness, higher bone marrow adiposity, higher levels of bone morphogenetic protein, and enhanced osteoblastogenesis. Retinaldehyde accumulates in retinaldehyde dehydrogenase‐deficient mice, and in vitro it strongly induces bone morphogenetic protein 2 (BMP2) (Nallamshetty et al., 2013). Given that vA intake is a known risk factor for osteoporosis and fractures (Melhus et al., 1998), these findings in mice deficient in retinaldehyde dehydrogenase suggest that in a largely parasite‐free and vA‐rich dietary environment, lower retinaldehyde dehydrogenase enzyme activity could lead to an accumulation of vA and to increased risks of osteoporosis, fractures, and other vA toxicity‐associated diseases. This could explain the fact that older African American women, despite having higher bone mineral densities (BMD) than EA women throughout life, are at significant risk of developing osteoporosis and of having poorer outcomes following diagnosis.

In patients with colorectal cancer, research on drug metabolism suggests the hypothesis that lower dehydrogenase enzyme activity leads to an accumulation of vA to toxic concentrations in the intestines and promotes disease pathogenesis. Dihydropyrimidine dehydrogenase (DPD) is the initial and rate‐limiting enzyme of 5‐fluorouracil (5‐FU), one of the most commonly used drugs to treat cancer. Studies have linked reduced DPD activity to the development of 5‐FU toxicity (i.e., leukopenia and anemia) and African Americans with colorectal cancer have increased 5‐FU–associated toxicity and decreased overall survival compared to EA patients (Mattison et al., 2006; Snyder et al., 2021). DPD enzyme activity in healthy AA volunteers is significantly reduced compared to healthy EA volunteer controls, with threefold higher rates of DPD deficiency in AAs compared to EAs (8.0% vs. 2.8%, respectively; *p* = 0.07) (Mattison et al., 2006).

## 13 IMPLICATIONS FOR PREVENTION

The adoption of traditional or modified plant‐based West Central African‐style diets that are low in preformed vA and high in vegetable fiber (Haskell, 2012; US Department of Agriculture, 2014) is proposed for study as an individual and population‐based strategy for improving health outcomes in African Americans, and also West Central Africans as the latter increasingly switch to western dietary practices. Dietary fiber refers to nondigestible carbohydrates and lignin, which are intrinsic and intact in plants, and its consumption lowers the risk of CVD, type 2 diabetes, and some cancers (Dahl & Stewart, 2015). Sources of fiber in these diets are cassava, millet, barley, sorghum, various legumes, fruits, and vegetables (USAblog). Dietary fiber interacts with the antioxidants in food and interferes with their assimilation. Soluble dietary fiber in the gut reduces the absorption of dietary fats and inhibits the absorption of carotenoids as lipid soluble compounds (Palafox‐Carlos et al., 2011). Increased consumption of dietary fiber could therefore contribute importantly to reducing the bioavailability of vA.

The dramatic impact of diet on people of African descent was shown in a study by O'Keefe and colleagues. Twenty AAs and 20 rural South Africans switched diets for two weeks. The Africans consumed American food, consisting mainly of meat and cheese. The AAs consumed the African high‐fiber diet. Africans on the American diet quickly developed intestinal mucosal inflammation and a cytokine profile in the gut related to intestinal cancer. In contrast, when the AAs were placed on a traditional African diet their existing intestinal inflammation disappeared whereas butyrate, a fatty acid known to protect against colon cancer, increased (O'Keefe et al., 2015). These effects were interpreted by O'Keefe as suggesting a potential toxin in the US diet to which people of West Central African descent are especially sensitive. The toxin in question may be vA.

## 14 TESTING THE THEORY

Studies to test the theory include: (1) comparing vA concentration profiles (including serum retinol, retinyl esters, retinoic acid, the ratio of retinyl esters to total vA, and rates of vA metabolism and catabolism) in healthy AAs, EAs and Americans of other ancestral origins; and (2) comparing vA profiles in AA and other patients with diseases for which AAs are at higher risk, including organ‐specific diseases and conditions. AA patients with these diseases are expected to be more likely than other patients to have lower concentrations of retinol but increased retinyl ester concentrations, higher percent retinyl esters to total vA, and higher retinoic acid concentrations. AA patients are also predicted to have increased retinyl esters and retinoic acid concentrations and receptor expression in the affected tissues, and lower rates of vA metabolism and catabolism. Studies are also indicated to determine the impact of interventions designed to optimize vA profiles in AA patients with diseases and conditions in which such alterations are identified.

## 15 CONCLUSIONS

The centuries‐long legacy of enslavement, oppression, discrimination, and stress suffered by African Americans continues to have pervasive adverse effects, including access to healthcare and risks of disease. Structural racism and discriminatory practices remain major targets for political action, advocacy, research, and health policy (Braveman & Gottlieb, 2014; Colen et al., 2017; deShazo, 2018). Less well known or understood is that people of West Central African descent are not only at increased risk of certain diseases but also of reduced risk of other diseases. Here the hypothesis is proposed that the overall health profile of people originally from this region of Africa is related to evolutionary adaptations to the ancestral environment, which involved continuous exposure to endemic tropical diseases. The central challenge of that environment is the major malaria parasite *Plasmodium falciparum* and other disabling parasites, which selectively absorb vitamin A from the human host, where its use in reproduction is also linked to disease pathogenesis. These adaptations evolved from natural selection and included (1) sequestering vA away from the liver to other organs, to reduce accessibility to the invaders, and (2) slowing the metabolism and catabolism of vitamin A, causing it to accumulate to subtoxic concentrations and weaken the parasite, thereby reducing disease severity. These adaptations were apparently successful in the original environment. However, in the North American environment, characterized by the absence of vA‐absorbing parasites and a dairy‐based, vA‐containing diet, people of West Central African descent may be especially susceptible to the vascular, metabolic, mutational, and allergic diseases in which vA toxicity is being found to play a major role. Subject to testing, the hypothesis suggests that the adoption of traditional or modified West Central African‐style diets that are low in vA and high in vA‐absorbing fiber hold promise for the prevention and treatment of these diseases and could also serve as a population‐based strategy for maintaining overall health and increasing longevity.

## CONFLICT OF INTEREST STATEMENT

None.

## Data Availability

Data sharing is not applicable to this article as no new data were created or analyzed in this study.
